# Complete chloroplast genome of *Pluchea indica* (L.) Less. (Asteraceae) and its phylogenetic analysis

**DOI:** 10.1080/23802359.2017.1413299

**Published:** 2017-12-07

**Authors:** Ying Zhang, Jingwen Zhang, Yong Yang, Qiang Liu

**Affiliations:** Key Laboratory of Tripical Animal and Plant Ecology, Ministry of Education, College of Life Science, Hainan Normal University, Haikou, China

**Keywords:** Chloroplast genome, illumine sequencing, *Pluchea indica* (L.) Less, phylogenetic analysis

## Abstract

This study presents the chloroplast genome of *Pluchea indica*(L.) Less, Asteraceae, one wide distributed species of mangrove associates plant in China, which was assembled and analyzed by de novo assembly using whole-genome sequencing data. The accessing NC_015621 was used as a reference sequence in this study. The size of the complete chloroplast genome was found to be 152,298 bp in length, comprising a large single copy region (LSC) of 84,127bp, a small single copy region (SSC) 18,068 bp, and inverted repeat regions (IRS) of 25,051bp. A total of 128 genes, including 84 protein-coding genes, 36 tRNA genes and eight rRNA genes, were predicted from the chloroplast genomes. Among them, 17 genes occur in IRS, containing six protein-coding genes, seven tRNA genes and four rRNA genes. The GC content of *P. indica* is 37.49%. The phylogenetic analysis with four Asterids species and five other species revealed that *P. indica* was clusted with *Ageratina adenophora*.

*Pluchea indica* (Asteraceae) is a perennial shrub plant indigenous in Indian and many Asian countries (Sen et al. 2002) and it is wide spread in the pacific islands as an introduced and often invasive species (Zeng et al. 2012). With the higher ability of salt tolerance and often grows in wet saline coastal habitat, it is classified as one mangrove associates species in China (Jin et al. 2017). The high medicinal values were researched in many countries as the traditional medicinal plant (Boonruang et al. 2017; Buranasukhon et al. 2017). The leaf extracts possess inhibitory activity against intestinal maltase. Enzyme assay-guided fractionation by chromatography yielded five active caffeoylquinic acid derivatives (Arsiningtyas et al. 2014). Methannolic extracts were identified having the anti-inflammatory properties and aqueous extracts having anti-cancer property of *P. indica* (Sen and Nag Chaudhuri 1991; Cho et al. 2012).

In the study, the sample was collected in Dongzhai Harbor, Haikou, China (N19°51′–20°1′, E110°32′–110°37′). Total DNA was isolated from fresh leaves of an individual of *P. indica*, which was deposited at the botany laboratory of Hainan normal University, Haikou, China, by using a DNeasy Plant Mini kit (Qiagen, Hilden, Germany) according to the instructions from the manufacturer. Genome sequencing was performed on an Illumina Hiseq X Ten platform (Illumina, San Diego, CA) with paired-end reads of 150 bp. In total, 1.591 Mb short sequence data with Q20 was 96.76% were obtained. Low-quality reads were filtered out and the remaining high-quality reads were used to assemble the chloroplast genome in SOAPdenovo (Luo et al. 2015). The genes in the chloroplast genome were annotated using the DOGMA program (Wyman et al. 2004). The circular chloroplast genome map was drawn using CpGAVAS software (Liu et al. 2012). The complete chloroplast genome was submitted to GenBank under the accession numbers of MG452144.

The *P. indica* chloroplast genome is 152,298 bp in length, with a pair of IR regions of 25,051 bp that separate an LSC region of 84,127 bp and an SSC region of 18,068 bp. It has a GC content of 37.49% and contains 128 protein-coding genes, 36 tRNA genes and eight rRNA genes. Among them, 17 genes occur in IRs, containing six protein-coding genes (*ndhB*, *rpl2*, *rpl23*, *rps7*, *rps12* and *ycf2*), seven tRNA genes (*trnl-CAU*, *trnL*-*CAA*, *trnV*-*GAC*, *trnl*-*GAU*, *trnA*-*UGC*, trnR-*ACG* and *trnN*-*GUU*) and four rRNA genes (*rrn4.5S*, *rrn5S*, *rrn16S* and *rrn23S*). Nine genes containing intron were found. Among them, seven genes contain one intron and one gene (*ycf3*) contains two introns. Interestingly, the gene (*clpP*) was found having three introns.

The phylogenetic relationship of it was studied with nine previously reported complete chloroplast genomes were downloaded for analysis, in which, four species were chosen in Asterids. Maximum-likelihood (ML) phylogenetic tree of nine species with rice as an outgroup (Figure 1). Those chloroplast genome sequences were aligned using MAFFT (Katoh and Standley 2013). Phylogenetic analysis using the maximum-likelihood algorithm was conducted with RAxML (Stamatakis 2014) implemented in Geneious ver. 10.1 (http://www.geneious.com, Kearse et al. 2012). The *P. indica* chloroplast genome provides essential and important DNA molecular data for phylogenetic and evolutionary analysis for this important traditional medical mangrove associates species.

**Figure 1.**
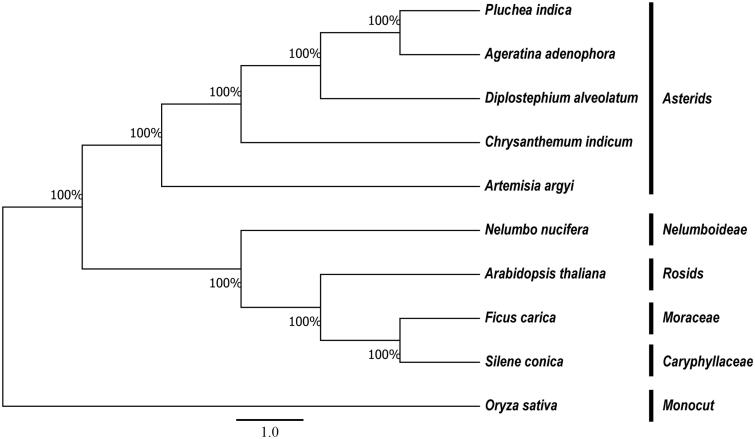
Phylogenetic relationship of the *P. indica* chloroplast genome with twelve previously reported complete chloroplast genomes. Numbers in the nodes are the bootstrap values from 1000 replicates.
